# Why don't poor men eat fruit? Socioeconomic differences in motivations for fruit consumption^☆^

**DOI:** 10.1016/j.appet.2014.10.022

**Published:** 2015-01-01

**Authors:** Rachel Pechey, Pablo Monsivais, Yin-Lam Ng, Theresa M. Marteau

**Affiliations:** a Behaviour and Health Research Unit, Institute of Public Health, University of Cambridge, Cambridge CB2 0SR, United Kingdom; b Centre for Diet and Activity Research, University of Cambridge School of Clinical Medicine, Box 285 Institute of Metabolic Science, Cambridge CB2 0QQ, United Kingdom

**Keywords:** Socioeconomic status, Liking, Motivation, Fruit, Consumption

## Abstract

*Background:* Those of lower socioeconomic status (SES) tend to have less healthy diets than those of higher SES. This study aimed to assess whether differences in motivations for particular foods might contribute to socioeconomic differences in consumption. *Methods:* Participants (n = 732) rated their frequency of consumption and explicit liking of fruit, cake and cheese. They reported eating motivations (e.g., health, hunger, price) and related attributes of the investigated foods (healthiness, expected satiety, value for money). Participants were randomly assigned to an implicit liking task (Single Category Implicit Association Task) for one food category. Analyses were conducted separately for different SES measures (income, education, occupational group). *Results:* Lower SES and male participants reported eating less fruit, but no SES differences were found for cheese or cake. Analyses therefore focused on fruit. In implicit liking analyses, results (for income and education) reflected patterning in consumption, with lower SES and male participants liking fruit less. In explicit liking analyses, no differences were found by SES. Higher SES participants (all indicators) were more likely to report health and weight control and less likely report price as motivators of food choices. For perceptions of fruit, no SES-based differences were found in healthiness whilst significant interactions (but not main effects) were found (for income and education) for expected satiety and value for money. Neither liking nor perceptions of fruit were found to mediate the relationship between SES and frequency of fruit consumption. *Conclusions:* There is evidence for social patterning in food motivation, but differences are modified by the choice of implicit or explicit measures. Further work should clarify the extent to which these motivations may be contributing to the social and gender patterning in diet.

## Highlights

• The study examined intake, implicit and explicit liking, and perceptions of fruit.
• Lower SES males reported eating less fruit and lower implicit liking of fruit.
• Results differed for explicit liking of fruit, however, with no differences by SES.
• Social patterning was also seen in perceived satiety and value for money of fruit.
• Neither liking nor perceptions of fruit mediated social patterning of fruit intake.

## Introduction

There are substantial socioeconomic inequalities in the prevalence of non-communicable diseases including diabetes, cardiovascular disease and cancer, the key determinants of which are behavioural risk factors, including unhealthy diets (World Health Organization, 2011). The consumption of unhealthy diets (in particular, eating fewer fruits and vegetables) is also strongly patterned by socioeconomic status (SES) (Appelhans et al, 2012, Darmon, Drewnowski, 2008, Giskes et al, 2010, Pechey et al, 2013, UK Department for Environment, Food and Rural Affairs, 2011).

Population-level factors implicated in the association between SES and fruit and vegetable consumption include food environments, with those in lower SES groups having less physical access to healthier food outlets and greater exposure to unhealthy outlets (Cummins et al, 2009, Molaodi et al, 2012, Smith et al, 2010), and economic access, with more energy-dense foods often providing cheaper sources of calories (Monsivais, Mclain, & Drewnowski, 2010). These socioeconomic differences in fruit and vegetable consumption may also be influenced by cross-cultural differences, with large purchasing gaps by SES observed for fruit in the UK, Belgium and Germany, but non-significant differences in Sweden, Italy and Spain (Fernández-Alvira et al, 2013, UK Department for Environment, Food and Rural Affairs, 2011).

Numerous individual-level factors have also previously been identified as partial mediators of the relationship between socioeconomic status and diet quality, including: attitudes to healthy eating (Ball et al, 2006, Le et al, 2013), nutrition knowledge (Ball et al, 2006, McLeod et al, 2011), stressors and psychological resources (Mulder, de Bruin, Schreurs, van Ameijden, & van Woerkum, 2011), diet cost (Aggarwal, Monsivais, Cook, & Drewnowski, 2011) and higher consumption of takeaways (leading to lower fruit and vegetable consumption) (Miura, Giskes, & Turrell, 2011).

Another individual-level factor, food motivation – defined as the extent to which participants value a particular food in comparison to other food items or to non-food alternatives – was identified in a recent review as one of the most reliable neurobehavioural correlates of obesity, associated with a range of food-related behaviours (Vainik, Dagher, Dubé, & Fellows, 2013). One factor in determining motivation is liking for different foods, which may be socially patterned – e.g., lower compared with higher income groups report greater dislike for healthier versions of selected foods including wholemeal bread, rice and pasta, low fat yoghurt and unsweetened fruit juice (Turrell, 1998). Limited evidence exists on the social patterning of motivation towards different foods, however – this has mostly been generated using explicit, self-report measures of liking. Additionally investigating implicit measures of liking is of interest, given these have previously been suggested to predict impulsive rather than controlled behaviour (Friese et al, 2008, Hofmann et al, 2007).

As well as liking, several other diet-related motivations have been shown to influence food choice, reflected in measures developed to assess the range of eating motivations (Eating Motivations Survey; Food Choice Questionnaire: Renner et al, 2012, Steptoe et al, 1995). In particular, studies looking at the importance given to price and health considerations consistently reveal socioeconomic disparities (Bowman, 2006, Konttinen et al, 2013, Steptoe, Wardle, 1999). Individuals would be expected to choose foods according to their reported eating motivations where possible. Disparities in eating motivations could therefore be exacerbated by differences in nutritional knowledge, but whilst SES differences in knowledge have been shown at an aggregate level (Ball et al., 2006), the consistency of perceptions (e.g., healthiness) of particular foods across socioeconomic groups has not been investigated to our knowledge. Beyond cost and health considerations of different types of foods, the perceived satiety of these foods is of interest, given that satisfying hunger is a primary motivation to eat, and of particular importance for those with limited resources for purchasing food.

In this study, we tested the hypothesis that social patterning for food motivation, using both implicit and explicit measures of liking, reflects the social patterning observed in food choices. In addition, we investigated social patterning in usual eating motivations and related perceived attributes of the investigated foods. The aim was to assess the extent to which differences in food motivations might contribute to socioeconomic differences in food choice, with possible implications for policies aimed at reducing the social patterning of diet quality and subsequent health inequalities.

## Material and methods

### Sample and design

Seven hundred thirty-two members of an online panel (aged 18+) were recruited via a research agency, with interlocking quotas set for occupational group and gender. To be eligible, participants had to pass quality control check questions (e.g., participants had to respond correctly after certain questions when the following message was shown: *“(PLEASE NOTE: This question is to check that you are reading questions carefully. Please ignore the question above, and instead select ‘other’, and write the word ‘X’)”*). Participants completed the study online, on their own computers.

A mixed between- and within-design was used for the study: all participants completed the majority of measures but participants were randomly allocated to complete tasks with a high response burden (the Single Category Implicit Association Task) for just one of the three food categories under investigation. Sample size calculations suggested a total sample size of 738 to detect differences in food motivation (including the Single Category Implicit Association Task) by socioeconomic status and gender (interactions) in each food category (for power of 0.8, α of 0.05, and an effect size of 0.2, based on Lien, Jacobs, & Klepp, 2002).

### Measures

#### Socioeconomic status

A range of indices to assess different aspects of socioeconomic status were collected:

(1) occupational classification of the respondent, using the UK Registrar General's social classification (Rose & Pevalin, 2001), categorised into three groups: A&B: higher managerial and professional; C1&C2: white collar and skilled manual; and D&E: semi-skilled and unskilled manual
(2) total household income before tax (categorised into four groups of roughly equal sizes, recoded from initial responses where participants selected from 15 income bands)
(3) highest educational qualification (questions and categorisations used were in line with the approach in the 2011 UK census, but combining ‘no qualifications’ and ‘1–4 GCSEs or equivalent’ groups due to the low number of respondents falling into the ‘no qualifications’ category, giving four groups): ‘No qualifications, GCSE D–G grades, or Level 1 NVQ’; ‘GCSE A*–C grades, or Level 2 NVQ’; ‘A/AS level, or Level 3 NVQ’; ‘Degree or Professional Diploma’^1^

#### Other participant characteristics

Data on gender, age, ethnicity, self-reported height and weight (used to calculate participants' body mass index: BMI), current hunger (measured via a 7-point scale, from ‘Very hungry’ to ‘Very full’), and the number of adults and children living in their household (used to calculate a household composition equivalence score (Organisation for Economic Co-operation and Development-modified scale): households score 1 for the respondent, 0.5 for each additional adult and 0.3 for each child) were also collected.

#### Food categories

The three categories – fresh fruit, cheese and cake – were selected as categories which have previously been observed to be social patterned (Pechey et al., 2013), that all require little preparation prior to consumption, and that reflect a range of healthiness.

#### Frequency of consumption

Participants were asked to indicate how often they consumed cheese, fruit and cake (along with a selection of other ‘filler’ food items) using the categories: ‘Every day’; ‘4–6 times a week’, ‘2–3 times a week’, ‘Once a week’, ‘Less than once a week’ and ‘Never’.

#### Implicit Liking: Single Category Implicit Association Task

Implicit liking was operationalised as the differential association between the attribute ‘I like it’ and pictures of food items vs. the attribute ‘I don’t like it’ and the pictures of food items for each food category, using the SC-IAT procedure described by Karpinski and Steinman (2006). For each food category, a set of eight pictures was selected from those freely available online (each equally sized, showing the food item by itself, against a white background). Each picture depicted a different exemplar food from the category (e.g., for fruit, pictures showed apple, grapes, melon, banana, pineapple, orange, cherries, peaches). Participants were given a practice block of 24 trials that paired pictures of foods from the target category with either the attribute ‘I like it’ or ‘I don’t like it’ (following the terms used in Nederkoorn, Houben, Hofmann, Roefs, & Jansen, 2010; the same pictures were used in all blocks). This was followed by the corresponding full test block, which comprised 72 trials. The procedure was then repeated for the second attribute. In each trial stimuli were presented for a maximum of 1500 ms (with a pre-trial pause of 250 ms). If participants did not respond in this time, they saw a reminder to ‘please respond more quickly’. Participants received error feedback, but did not have to correct their responses to proceed.

Scores for the SC-IAT were based on the test blocks only. Responses of less than 350 ms or longer than 10 s were excluded from analysis, as were nonresponses. Error responses were replaced with the block mean plus a penalty of 400 ms (as in Karpinski & Steinman, 2006). Participants with an error rate of greater than 33% were excluded (n = 37). The average response times of the two trial blocks were subtracted from each other, and then divided by the pooled standard deviation of all correct response times within the two blocks. Higher scores indicate greater liking for foods (see Supplementary Table S2 for summary statistics).

#### Explicit Liking: Enjoyment of Consumption

Participants were presented with pictures of six foods from each of the three food categories and asked to use 7-point scales, labelled with the anchors ('Very unenjoyable'; ‘Unenjoyable’; ‘Quite unenjoyable’; ‘Neither enjoyable nor unenjoyable’; ‘Quite enjoyable’; ‘Enjoyable’; 'Very enjoyable'), to rate these individual foods for enjoyment of consumption. These items were coded with a score between −3 (‘Very unenjoyable’) and 3 (‘Very enjoyable’). Overall ratings were determined by calculating the mean rating given to the six pictures presented. Higher scores indicate greater liking.

#### Perceived attributes of food category

Participants were asked about their perceptions of the three foods presented in the study: fruit, cake and cheese. When participants were presented with pictures of foods from the three food categories, alongside with the explicit liking measure (described above), they were also asked to use 7-point scales to rate these individual foods for perceived healthiness, satiety and value for money (‘How healthy is this food?’, from ‘Very unhealthy’ to ‘Very healthy’; ‘How fast would you get hungry again after eating this food?’, from ‘Very slowly’ to ‘Very quickly’; ‘Is this food usually good value for money?’, from ‘Very poor value for money’ to ‘Very good value for money’). These items were coded with a score between −3 (‘Very unhealthy’/‘Very quickly’/‘Very poor value for money’) and 3 (‘Very healthy’/‘Very slowly’/‘Very good value for money’), with overall ratings again determined by calculating the mean rating given to the pictures presented. Higher scores indicate greater perceived healthiness, satiety or value for money.

### Eating motivations

Participants rated the frequency with which their usual food choices were influenced by each of a range of motivations (ratings from 1 ‘Never’ to 7 ‘Always’), using short-forms of selected factors (Liking, Habits, Need & hunger, Health, Convenience, Pleasure, Price, Weight control) from The Eating Motivations Survey (Renner et al., 2012). Motivation scores for each of the selected factors were determined by taking the mean rating across the questions comprising that subscale.

### Analyses

To determine whether there were socioeconomic differences in the frequency of consumption of investigated foods, proportional odds models were run. These analyses predicted frequency of consumption from key variables: dummies for socioeconomic indicator groups (analyses for each indicator being run separately), gender, and socioeconomic group by gender interactions. Age, hunger, BMI, ethnicity (white/other), and household composition were included as covariates.

For those foods that showed social patterning, socioeconomic differences in implicit and explicit liking, eating motivations, and perceived attributes of food categories, were examined using multiple regression analyses. Predictors and covariates were the same as in the frequency of fruit consumption analyses described above.^2^ Analyses on implicit liking comprised just those participants randomised to the fruit category (n = 229).

For implicit and explicit liking measures that were shown to be socially patterned, causal mediation analyses were conducted (using the ‘mediation’ command in R, as detailed in Imai, Keele, & Tingley, 2010) to assess whether liking acts as an intermediate variable, positioned in a causal pathway between socioeconomic status and frequency of consumption (i.e. ‘average causal mediation effects’). Similar analyses were run for those perceived attributes of food categories that were socially patterned. As the methodology for conducting moderated mediation with multinomial independent and dependent variables is currently not available to our knowledge, analyses were gender-stratified for those analyses for which SES and gender interactions were significant.

## Results

### Sample characteristics

The final sample consisted of 361 men and 371 women, with a mean age of 51 (s.d. 14, range 18–92), 95.5% of whom were of white ethnicity. In terms of the different socioeconomic indicators, the sample was, by design, close to evenly divided by occupational group (34% A&B [higher managerial and professional], 33% in C1&C2 [skilled manual], and 32% in D&E [semi-skilled and unskilled manual]) and household income (25% ‘Up to £15,499 per year’; 24% ‘£15,500–£24,999 per year’; 27% ‘£25,000–£39,999 per year’ and 23% ‘£40,000 or more per year’; with 2% ‘Don’t know/Prefer not to say’). For highest educational qualification, 41% of the sample fell into the highest category (‘Degree or Professional Diploma’), with roughly even percentages in the lower groupings (19% ‘No qualifications, GCSE D–G grades, or Level 1 NVQ’; 18% ‘GCSE A*–C grades, or Level 2 NVQ’; 17% ‘A/AS level, or Level 3 NVQ’; with 6% ‘Other/Prefer not to say’). This suggests the study sample is more educated than average, with 27% of the population in England and Wales reporting degree or higher education in the 2011 census (Office for National Statistics, 2014). See Supplementary Table S1, for more details on participant characteristics.

## Frequency of consumption

**Fig. 1:**
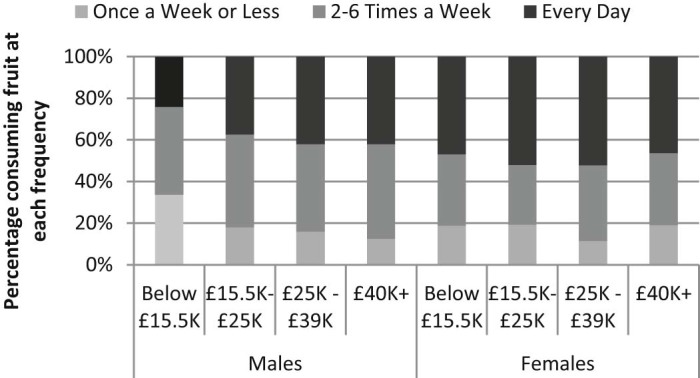
Frequency of fruit consumption by income group and gender.

Of the three food categories under investigation, only fresh fruit consumption was found to be socially patterned (see Fig. 1 for frequency of consumption of fruit; Supplementary Fig. S1 for frequency of consumption of cheese and cake). Frequency of fruit consumption was categorised as high (every day), medium (2–6 times a week), or low (once a week or less), with higher proportions of those in higher compared to lower income groups reporting high fruit consumption (odds ratios of 2.06, 2.67 and 2.86 for groups with incomes of £15,000–£25,000 pa, £25,000–£39,000 pa, and £40,000 + pa, respectively, relative to those with incomes up to £15,500 pa; 95% CIs: 1.16–3.65, 1.54–4.63, 1.58–5.15). This paper therefore focuses on explaining the social patterning of consumption within this food category.

Results are presented first by income group, with any differences depending on choice of socioeconomic indicator then being highlighted where evident. The most deprived socioeconomic group and males are always taken as the reference group(s).

**Fig. 2:**
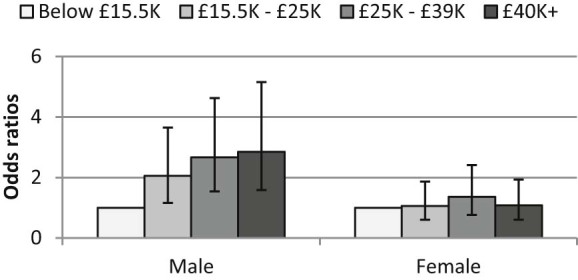
Probability of eating fruit daily (odds ratios), by income group and gender. N.B. Presented odds ratios are gender-specific; CIs for females were calculated by re-running models with females as the reference group.

Figure 2 shows the odds ratios determined by the proportional odds model for frequency of fruit consumption (see Table 1 for full results). For example, the odds of reporting high-frequency fruit consumption rather than medium- or low-frequency consumption are 3.13 times (95% CI 1.78–5.51) greater for women vs. men (all other variables being held constant). In this model, these odds are assumed to be the same when comparing those reporting high- or medium-frequency fruit consumption to those reporting low-frequency consumption – that is, the model assumes that the relationship between low vs. medium/high consumption is the same as the relationship between low/medium vs. high consumption (and tests suggest this assumption holds for the models investigated).

**Table 1:** Proportional odds model (frequency of fruit consumption by income group).

		Odd ratios (95% CI)
Household income (£1000/year)	*15.5–25*	**2.06 (1.16, 3.65)**
	*25–39*	**2.67 (1.54, 4.63)**
	*40+*	**2.86 (1.58, 5.15)**
Gender	*Female*	**3.13 (1.78, 5.51)**
Income × Gender interaction terms	*15.5–25 Female*	0.52 (0.23, 1.15)
	*25–39 Female*	0.51 (0.23, 1.12)
	*40+ Female*	**0.38 (0.17, 0.85)**
Age		**1.03 (1.02, 1.05)**
Hunger rating		1.01 (0.91, 1.13)
BMI		**0.97 (0.95, 1.00)**
Ethnicity	*White*	1.11 (0.56, 2.20)
Household composition		0.97 (0.72, 1.35)
*No of observations*		707
*Likelihood ratio*^a^		0.02

Results significant at p < 0.05 in bold.

^a^The likelihood ratio compares coefficients from proportional odds model to those from a multinomial logit model with same variables; results suggest that the proportional odds model is a better fit than the equivalent multinomial logit model.

Figure 2 shows odds ratios for each income–gender group, reflecting an interaction whereby the odds of eating fruit more frequently are 2.9 times greater (95% CI 1.58–5.15) for men with incomes of £40,000 + pa vs. men with incomes up to £15,500 pa, but there were no significant differences between women in with incomes of £40,000 + pa and women with incomes up to £15,500 pa (with a significant interaction term for those with incomes of £40,000 + pa by gender (odds ratio 0.38, 95% CI 0.17–0.85); all other variables being held constant).

This interaction did not reach significance for other socioeconomic indicators (see Supplementary Fig. S2 for odds ratios for frequency of fruit consumption by the other SES indicators and gender). Using other SES indicators, main effects of SES were found for separate analyses with education and occupational group, showing a similar pattern to that for income. For education, significant differences were found between those whose highest qualifications were GCSEs at grades D–G and each of the higher educational groups (odds ratios of 2.12, 2.19 and 3.10 for those with (i) GCSEs at grades A*–C, (ii) A/AS levels, and (iii) Degrees or higher, respectively, relative to GCSEs at grades D–G; 95% CIs: 1.09–4.11, 1.14–4.21, 1.78–5.37). For occupational group only the highest group (A and B) showed a significant difference in fruit consumption frequency relative to the lowest group (D and E) (odds ratio 2.53, 95% CI 1.57–4.11).

## Implicit and explicit liking

### Implicit liking

**Fig. 3:**
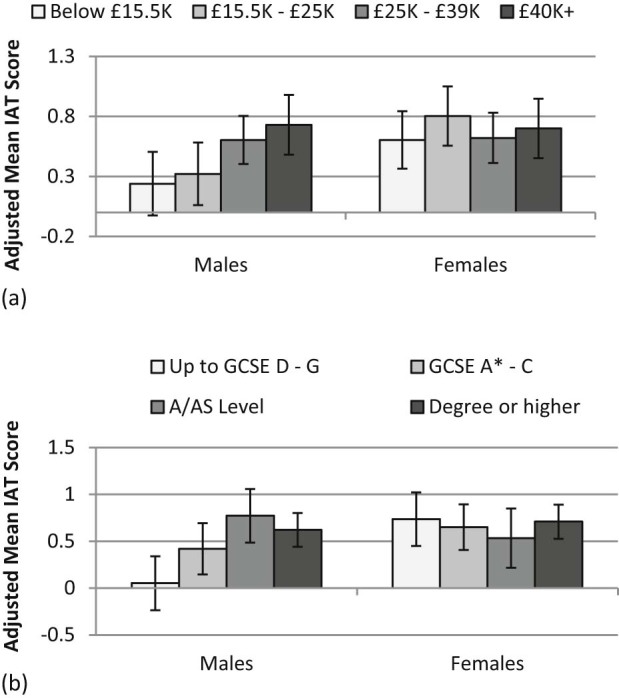
Implicit attitudes (IAT score) by (a) income group and gender and (b) education group and gender (adjusted means, with 95% CIs; for observed score range −1.6 to 2.5).

Those in the highest income group (£40,000 + pa) had average implicit liking scores for fruit that were 0.49 units (95% CI 0.04–0.94) higher than those in the lowest income group (Up to £15,500 pa) (mean implicit liking score 0.58, s.d. 0.63), i.e. those in the highest income group had higher implicit liking for fruit compared to those in the lowest income group. Whilst for the income analyses, SES and gender interactions did not reach significance, these interactions were significant for education (with a main effect of education such that those with A/AS levels or Degrees or higher had significantly higher implicit liking for fruit than those with GCSEs at grades D–G (coefficients of 0.72 and 0.57, with 95% CIS of 0.29–1.15 and 0.18–0.96, respectively); a main effect of gender with females having an average score 0.68 units higher than males (95% CI 0.21–1.16); and significant interaction effects for those with A/AS levels by gender (coefficient: −0.92, 95% CI: −1.54 to −0.31) and those with Degrees or higher by gender (coefficient: −0.60, 95% CI: −1.14 to −0.06)). Figure 3 suggests that whilst for females, education did not influence implicit liking for fruit, for males, lower levels of education may be associated with less liking for fruit.

For analyses examining occupational group, gender did not reach significance as a predictor of implicit liking, and no clear linear associations (main effects or interactions) were observed for occupational group and implicit liking.

### Explicit liking

Overall, all participants tended to report enjoying consuming fruit regardless of SES group, with women reporting higher levels of enjoyment than men (see Table 2: females having higher scores by between 0.24 and 0.28 (95% CI: lower bound between 0.10 and 0.15; upper bound between 0.37 and 0.42), for income, education and occupational group analyses without interactions, as these were all non-significant). There were no socioeconomic differences in ratings of enjoyment of consuming fruit using any of the SES indicators.

**Table 2:** Linear regression b coefficients (95%CIs): Implicit and explicit liking and perceived attributes of fruit, by income group.

		Implicit liking	Explicit liking	Perceived healthiness	Perceived satiety	Perceived value for money
Household Income (£1000/year)	*15.5–25*	0.08 (−0.32, 0.49)	−0.01 (−0.29, 0.27)	−0.04 (−0.25, 0.15)	**0.27 (0.03, 0.51)**	−0.01 (−0.30, 0.27)
	*25–39*	0.37 (−0.03, 0.76)	−0.03 (−0.30, 0.24)	−0.03 (−0.22, 0.17)	0.06 (−0.18, 0.29)	0.16 (−0.11, 0.43)
	*40+*	**0.49 (0.03, 0.95)**	0.02 (−0.26, 0.31)	0.00 (−0.19, 0.20)	**0.37 (0.13, 0.61)**	0.17 (−0.10, 0.44)
Gender	*Female*	0.36 (−0.07, 0.81)	**0.29 (0.03, 0.56)**	**0.22 (0.03, 0.42)**	**0.33 (0.09 0.57)**	**0.34 (0.04, 0.63)**
Income × Gender interaction terms	*15.5–25 Female*	0.12 (−0.41, 0.64)	0.03 (−0.33, 0.39)	0.11 (−0.16, 0.38)	−0.30 (−0.67, 0.07)	0.03 (−0.36, 0.42)
	*25–39 Female*	−0.35 (−0.86, 0.16)	0.06 (−0.30, 0.43)	0.01 (−0.25, 0.27)	−0.05 (−0.40, 0.30)	−0.19 (−0.58, 0.19)
	*40+ Female*	−0.39 (−0.96, 0.17)	−0.16 (−0.55, 0.23)	−0.11 (−0.37, 0.15)	−**0.55 (**−**0.91,** −**0.20)**	−**0.48 (**−**0.87,** −**0.09)**

Lowest household income group (up to £15,500) was the reference group; results significant at p < 0.05 in bold).

## Perceived attributes of food category

**Fig. 4:**
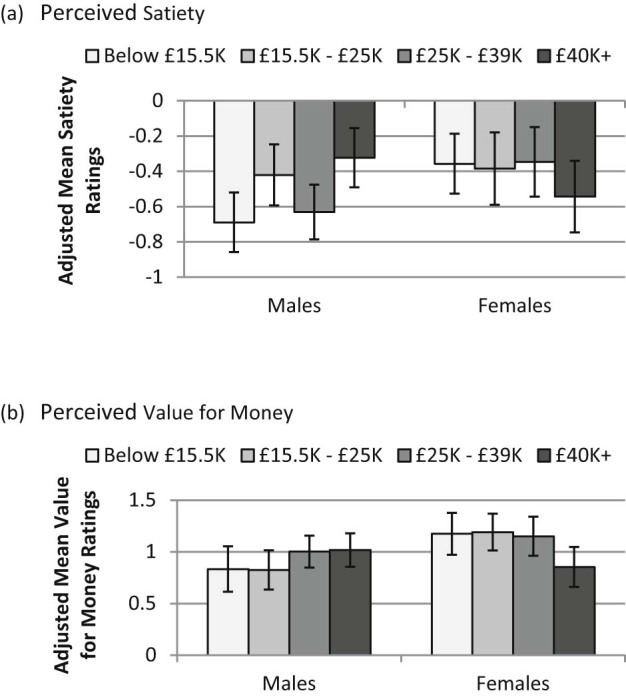
Ratings of (a) perceived satiety and (b) perceived value for money, by income group and gender (adjusted means, with 95% CIs; possible score range −3 to 3).

There were no socioeconomic differences by income in perceived healthiness of fruit (see Supplementary Table S2 for summary statistics; Supplementary Table S3 for correlations between implicit and explicit ratings and perceived attributes). However, there were significant interactions for perceived satiety and value for money, such that, if social patterning was evident, ratings by women tended to decrease with increasing income, whilst for men, ratings tended to increase with increasing income (Fig. 4).

Similar results were found for analyses looking at education rather than income; there were no main effects for education on healthiness ratings. The highest educational group (degree or higher) was a significant predictor of satiety ratings (coef: 0.32, p < 0.01), and a trend towards the interaction between the highest educational group and being female as a significant predictor of satiety ratings (Coefficients: Gender: −0.26; Degree or higher by gender: 0.33; p = 0.07) (Supplementary Fig. S3). For value for money ratings, there was no main effect of education, but again there was a trend towards the interaction between highest educational group and being female as a significant predictor of value for money ratings (coefficients: Degree or higher = 0.01; Gender = 0.46; Degree or higher by gender = −0.36; p = 0.06) (Supplementary Fig. S3).

For occupational group, no significant effects were found for any of the perceived attribute ratings (Supplementary Fig. S3).

## Eating motivations

Table 3 shows the results of regression analyses investigating socioeconomic differences in eating motivations, finding significant differences by household income group for Health, Convenience, Price and Weight control. No interactions were found, so the analyses in Table 3 are reported for regressions run without interaction terms.

**Table 3:** Linear regression b coefficients (95%CIs): Eating motivations by income group.

		Health	Convenience	Price	Weight control
Household Income (£1000/year)	*15.5–25*	−0.03 (−0.26, 0.20)	−0.17 (−0.38, 0.04)	−**0.24 (**−**0.47,** −**0.00)**	0.07 (−0.18, 0.33)
	*25–39*	0.16 (−0.07, 0.39)	−**0.21 (**−**0.39,** −**0.02)**	−**0.49 (**−**0.71,** −**0.27)**	**0.38 (0.12, 0.64)**
	*40+*	**0.24 (0.00, 0.47)**	−0.16 (−0.35, −0.04)	−**0.71 (**−**0.93,** −**0.50)**	0.27 (−0.01, 0.54)
Gender	*Female*	**0.56 (0.40, 0.72)**	0.05 (−0.09, 0.18)	0.09 (−0.06, 0.25)	**0.73 (0.54, 0.91)**

Lowest household income group (up to £15,500) was the reference group; results significant at p < 0.05 in bold.

Participants in higher income groups compared with the lowest income group rated health and weight control as more frequently acting to motivate their food choices, and rated price and convenience as less frequent motivations.

Analyses using alternative socioeconomic indicators showed that for education similar patterns held for health, price and weight control (although for weight control, an interaction between gender and education level was found). No social patterning in ratings for convenience was found for education, but participants with higher compared with the lowest levels of education rated habit (coefficients of −0.29, −0.31 and −0.33 for those with GCSEs at grades A*–C, A/AS levels, or Degrees or higher, relative to those with GCSEs at grades D–G, respectively; 95% CIs: −0.49 to −0.09, −0.51 to −0.10, and −0.50 to −0.16) and pleasure (coefficients of −0.25 and −0.20 for those with A/AS levels, or Degrees or higher, relative to those with GCSEs at grades D–G, respectively; 95% CIs: −0.47 to −0.04, and −0.37 to −0.02) as less frequently motivating their food choices. Analyses by occupational group revealed the same patterns as for income.

### Mediation analyses

Non-parametric bootstrap mediation analyses were conducted to examine whether implicit liking and perceived attributes of food categories (those that varied by SES), mediated the relationship between SES and frequency of fruit consumption. Mediation analyses were conducted separately for each SES indicator that showed significant relationships with the attitude variables.

For implicit liking, these analyses were limited to those individuals who completed the SC-IAT for fruit (n = 212 for income analyses). These analyses found no evidence that implicit liking acted as a mediator in these associations, for any of the socioeconomic indicators (Table 4).

**Table 4:** Mediation effects: Implicit liking and perceived attributes (as mediators of the relationship between income group and frequency of fruit consumption) (95% CIs in parentheses).

		Frequency of fruit consumption		
		Once a week or less	2–6 times a week	Every day
Implicit liking	Average Causal Mediation Effects	0.02 (−0.00, 0.05)	−0.00 (−0.02, 0.00)	−0.01 (−0.04, 0.00)
	Average Direct Effects	0.14 (−0.00, 0.25)	−0.05 (−0.07, 0.01)	−**0.09 (**−**0.19,** −**0.00)**
	Total Effect	**0.16 (0.01, 0.27)**	−0.05 (−0.07, 0.00)	−**0.11 (**−**0.20,** −**0.01)**
Perceived satiety	Average Causal Mediation Effects	0.00 (−0.00, 0.01)	−0.00 (−0.00, 0.00)	−0.00 (−0.01, 0.00)
	Average Direct Effects	**0.12 (0.05, 0.19)**	−**0.05 (**−**0.08,** −**0.02)**	−**0.06 (**−**0.12, 0.02)**
	Total Effect	**0.12 (0.05, 0.19)**	−**0.06 (**−**0.08,** −**0.02)**	−**0.07 (**−**0.12,** −**0.03)**
Perceived value for money	Average Causal Mediation Effects	−0.00 (−0.00, 0.00)	0.00 (−0.01, 0.01)	−0.00 (−0.01, 0.01)
	Average Direct Effects	**0.12 (0.04, 0.19)**	−**0.05 (**−**0.08,** −**0.02)**	−**0.06 (**−**0.11,** −**0.02)**
	Total Effect	**0.12 (0.04, 0.19)**	−**0.05 (**−**0.08,** −**0.02)**	−**0.06 (**−**0.11,** −**0.02)**

Results significant at p < 0.05 in bold.

For perceived attributes, no mediation was found between income and frequency of fruit consumption for either satiety or value for money. However, for education, perceived value for money showed inconsistent mediation of the relationship between SES and frequency of fruit consumption, but only for women (Table 5). As such, ratings of value for money (which for women were socially patterned such that those in higher SES groups perceived fruit as less good value for money, but vice versa for men) acted as a suppressor of the relationship between SES and frequency of fruit consumption in women (e.g., for eating fruit once a week or less, average causal mediation effect: −0.05; average direct effect: 0.12; total effect: 0.07).

**Table 5:** Mediation effects by gender of perceived value for money as a mediator of the relationship between education group and frequency of fruit consumption (95% CIs in parentheses).

		Frequency of fruit consumption		
		Once a week or less	2–6 times a week	Every day
Males	Average Causal Mediation Effects	−0.01 (−0.04, 0.03)	0.00 (−0.02, 0.03)	0.00 (−0.01, 0.02)
	Average Direct Effects	**0.20 (0.11, 0.28)**	−**0.19 (**−**0.30,** −**0.09)**	−0.01 (−0.04, 0.03)
	Total Effect	**0.19 (0.09, 0.28)**	−**0.18 (**−**0.29,** −**0.07)**	−0.01 (−0.03, 0.03)
Females	Average Causal Mediation Effects	−**0.05 (**−**0.09,** −**0.02)**	**0.02 (0.01, 0.04)**	**0.03 (0.01, 0.05)**
	Average Direct Effects	**0.12 (0.00, 0.23)**	−**0.07 (**−**0.18,** −**0.00)**	−**0.05 (**−**0.08,** −**0.00)**
	Total Effect	0.07 (−0.05, 0.20)	−0.04 (−0.13, 0.03)	−0.03 (−0.08, 0.03)

Results significant at p < 0.05 in bold.

## Discussion

Those in lower SES groups and men regardless of SES consistently reported eating fruit less frequently across all three socioeconomic indicators investigated. For income, gender modified the association between income and frequency of fruit consumption such that income was a stronger predictor for men. No socioeconomic differences were seen in terms of consumption of cheese or cake, but whilst associations have previously been found for these food categories using larger samples (Pechey et al., 2013), these were relatively small compared to the consistent differences found for fruit and vegetable purchasing or consumption (Appelhans et al, 2012, Darmon, Drewnowski, 2008, Giskes et al, 2010, Pechey et al, 2013, UK Department for Environment, Food and Rural Affairs, 2011). This suggests that the current study may not have had the power to detect differences, or the relative broad frequency categories used may not have been sufficiently nuanced to reveal differences in intake.

The interaction between income and gender predicting frequency of fruit consumption is in contrast to a recent systematic review of fruit consumption across Europe, which did not find evidence for SES and gender interactions (Giskes et al., 2010). It should be noted however, that the studies from the UK included in the review all used occupational group as their main measure of SES, and no interaction was found in results for occupational group in the current study. Given the differences in what different SES indicators represent (Turrell, Hewitt, Patterson, & Oldenburg, 2003) and in their relationships with other associated variables (e.g., income showed the strongest association with food price as a motivator of food choices), the choice of SES indicator may be important when exploring social patterning. Whilst household income and education largely behaved similarly in the current study analyses, analyses using occupational group as the SES indicator showed no socioeconomic differences in implicit liking or any of the perceived attributes of fruit. This ties in with previous studies suggesting that income, education and occupational group each show different associations with diet (Galobardes et al, 2001, Maguire, Monsivais, in press, Turrell et al, 2003). Income may reflect the economic resources available to individuals, whilst education may be a proxy for a range of factors such as knowledge or ability to use nutritional information, and occupational group could represent social networks amongst other factors.

This study looked at both implicit and explicit measures of liking, which have previously been suggested to predict impulsive and controlled behaviour respectively (Friese et al, 2008, Hofmann et al, 2007). In terms of participants' liking for fruit, lower SES was associated with lower implicit liking (for income and education, but not occupational group analyses), with this SES difference being stronger in men in the education analysis. For explicit liking of fruit, however, no clear associations were observed with SES. Whilst this social patterning reflected that seen for fruit consumption, implicit attitudes were not found to be a mediator of the relationship between SES and frequency of fruit consumption. Although implicit attitudes to foods have been found to predict food choices made within studies (e.g., Hollands, Prestwich, & Marteau, 2011), the outcome variable used in the current study – frequency of fruit consumption – is a generic measure of typical behaviour and as such may be unlikely to reflect occasions when impulsive behaviour is key. Additionally, fruit may not be a food category in which impulsive consumption is as likely to occur as with less healthy food categories, such as snacks, which have often been used in previous studies (e.g., Nederkoorn et al., 2010). As such, further exploration of the predictive value of implicit liking of fruit under conditions likely to lead to impulsive decision making (e.g., cognitive depletion) may be useful to establish the extent to which this plays a role in consumption decisions.

Regarding the role of the other motivations investigated, there were some clear differences by SES (all indicators): participants in higher, compared with lower, SES groups were more likely to rate health and weight control as stronger motivations for their food choices, and to rate price as less important. These disparities in the importance given to price and health considerations are consistent with socioeconomic differences in food choice priorities identified in previous research (Bowman, 2006, Konttinen et al, 2013). Of particular interest in the current study, however, was exploring how these motivations might act alongside perceptions of fruit (healthiness, satiety and value for money). As social patterning was seen in health considerations but not in ratings of fruit healthiness, healthy eating goals are more likely to contribute to socioeconomic differences in consuming fruit rather than beliefs about the healthiness of fruit per se. In contrast, whilst there were SES differences in ratings of the expected satiety from fruit (higher SES (income and education) groups and females being more likely to rate fruit as more satiating), there was no social patterning in being motivated to eat by need or hunger, and indeed this was reflected in finding no evidence of mediation in the relationship between SES and frequency of fruit consumption by satiety ratings. Finally, both price and ratings of the value for money of fruit were socially patterned (with women rating fruit as better value for money, and with the gap between genders increasing in lower SES (income and education) groups). One might hypothesise therefore that price-related motivations and perceptions could provide a double-hit with regard to SES differences in fruit consumption for men, but analyses did not find value for money ratings mediating this relationship for men. Mediation analyses did, however, indicate ratings of value for money acted as a suppressor of the relationship between SES and frequency of fruit consumption (for education) for women – i.e. the association of higher SES with higher frequency of fruit consumption was diminished by perceptions of fruit as being less good value for money in women of higher rather than lower SES.

### Strengths and weaknesses of the current study

This study is one of the first to investigate social patterning in both implicit and explicit measures of attitudes, and to evaluate the relative importance of liking in explaining the social patterning of fruit consumption. Including both implicit and explicit measures allows us to use these initial findings to make predictions on those scenarios where implicit rather than explicit liking may lead to social patterning in behaviour. In addition, by using a range of socioeconomic indicators, this study highlights the different conclusions that result from choosing different variables, and the importance of exploring the impact of measure choice.

However, there were some limitations to the current study: notably, the outcome measure for frequency of fruit consumption had rather broad categories, offering little nuance to distinguish between higher frequency consumers, which may have masked some of the variance. However, the general patterning seen in the current study reflects that in the Health Survey for England (HSE), where for men 50% in the lowest income group and 70% in the highest group reported eating fresh fruit on the day of the survey, whilst for women it was approximately 60% and 75% respectively (Health and Social Care Information Centre, 2010). The lower equivalent figures in our study (for men, 25% in lowest income group reported eating fruit daily, compared to 40% in highest income group; for women, approximately 45% in both the highest and lowest income groups) are likely to reflect differences in the questions asked (e.g., those who eat fruit 2–6 times a week are grouped separately in our study, a group that would overlap with those reporting eating fruit on a random day in the HSE). An additional concern is that, as participants completed the study in their homes, the extent to which participants were able to give the study their full attention is unknown (although quality checks were used to ensure that participants were engaging with the study). Finally, there were concerns that the study was likely to have been under-powered to detect the mediation effects investigated (Fritz & MacKinnon, 2007).

### Implications for future research

The evidence from the current study shows an association but does not offer a clear account of the role of food liking in socioeconomic differences in food choice, in particular given the evidence that greater exposure of adults to particular foods is associated with increased liking of these products (Crandall, 1985, Mattes, 1994, Pliner, 1982, Stein et al, 2003, Zellner et al, 1999). To further explore the nature of the relationship between fruit consumption and liking, experimental studies might explore the use of evaluative conditioning to increase positive attitudes towards fruit (Hollands et al., 2011) and compare this with interventions that focus only on increasing exposure to fruit. In addition, the variety of fruits consumed may also influence food liking, given that individuals show stimulus satiation to foods they consume regularly (Hetherington, Pirie, & Nabb, 2002), and evidence that a reduced variety of fruit is consumed by lower SES groups (Giskes, Turrell, Patterson, & Newman, 2002). If further evidence suggests a role for implicit and/or explicit liking in determining food choice, another avenue for research is to explore whether the role of motivation differs for healthier vs. less healthy foods; for example, given suggestions that implicit attitudes are a better predictor than explicit attitudes for impulsive decisions (Friese et al., 2008), if less healthy foods are more likely than healthier foods to be chosen impulsively, implicit attitudes may vary in their ability to predict choices of healthier or less healthy foods. Finally, future better-powered studies could explore the interaction between general eating motivations and related perceptions of different types of foods (e.g., motivation by cost and value for money of fruit) as mediators of food choice.

### Implications for policy

The mediation analyses conducted in this study suggest that approaches targeting fruit liking may have only limited impact on fruit consumption, although the conditions under which liking may influence behaviour merit further investigation. In terms of the perceived attributes of fruit, beliefs about the healthiness of fruit are not likely to be contributing to socioeconomic differences in consuming fruit (with all groups perceiving fruit as very healthy), and as such stressing the health benefits of fruit is unlikely to be effective at increasing fruit consumption in lower SES groups. Indeed, Beydoun and Wang (2008) show that nutrition knowledge and beliefs interact with education, such that those with the healthiest nutrition knowledge and beliefs show the strongest patterning of consumption by education (i.e. health nutrition knowledge may be necessary but not sufficient to overcome socioeconomic differences). Also, the results suggest establishing perceptions of fruit as being good value for money may have some benefit, in that such perceptions appear to be boosting consumption of fruit in women of lower socioeconomic status. Targeting men of lower socioeconomic status may be effective, particularly given recent successes in interventions targeting changes in behaviour in this population (Hunt et al., 2014). If a role of food liking in influencing consumption is established, exposure to a wide variety of fruits at low prices may be promising if it leads to increased sampling of these foods, thereby setting up a virtuous circle such that increased consumption increases liking (e.g., Stein et al., 2003), which in turn increases consumption (e.g., Nederkoorn et al., 2010).

### Conclusions

There is evidence for social patterning in motivation for food, but differences are modified by the choice of implicit or explicit measures. Further work should clarify the extent to which these motivations may be contributing to the social and gender patterning of diet quality. In particular, targeting implicit attitudes would shed light on the causal nature of the observed association between fruit consumption and implicit attitudes as well as providing a novel intervention to increase fruit consumption to reduce the social and gender gradient in healthy diets.

## Footnotes

2 In addition, as the implicit measure involved reaction times, participants' computers' display refresh rates were included in the analysis of implicit liking, to try and control for any potential differences due to computer speed. No significant effect of computer display refresh rate was found.

## Footnotes

1 GCSEs (General Certificate of Secondary Education) are usually taken at age 15–16 in the UK; AS Levels at age 16–17; A-Levels at age 17–18; NVQs (National Vocational Qualifications) are work-based awards, with levels approximately equating to academic qualifications in their category.
