# Supplementary material for: Why don't poor men eat fruit? Socioeconomic differences in motivations for fruit consumption
Source: Appetite. 2015 Jan 1;84:271–9. doi: 10.1016/j.appet.2014.10.022 (PMC4262578; doi:10.1016/j.appet.2014.10.022)
Supplement: Appendix S1 — Tables S1–S3 and Figs. S1–S3. [file mmc1.docx]

**Supplementary Materials:**

**Why don’t poor men eat fruit? Socioeconomic differences in motivations for fruit consumption**

Contents

[Table S1: Participant characteristics 2](#_Toc396220371)

[Table S2: Mean score (s.d.) for eating motivations, implicit and explicit liking and perceived attributes by income group and gender 3](#_Toc396220372)

[Table S3: Correlations between implicit and explicit liking and perceived attributes 4](#_Toc396220373)

[Figure S1: Frequency of consumption of (a) cake and (b) cheese by income group 5](#_Toc396220374)

[Figure S2: Probability (odds ratios) of consuming fruit daily, by (a) education group and gender, and (b) occupational group and gender 6](#_Toc396220375)

[Figure S3: Ratings of: (a) perceived satiety and (b) perceived value for money by education group (adjusted means, with 95% CIs; possible score range -3 to 3) 7](#_Toc396220376)

| Table S1: Participant characteristics  \|  \| \| **Frequency (%)** \| \| \| \| --- \| --- \| --- \| --- \| --- \| \| *Males* \| *Females* \| *Total* \| \| **Age** \| 18-39 \| 61 (17.4) \| 103 (28.0) \| 164 (22.8) \| \| 40-64 \| 211 (60.1) \| 200 (54.3) \| 411 (57.2) \| \| 65+ \| 79 (22.5) \| 65 (17.7) \| 144 (20.0) \| \| **Ethnicity** \| White \| 346 (95.8) \| 353 (95.4) \| 699 (95.6) \| \| Non-white \| 15 (4.2) \| 17 (4.6) \| 32 (4.4) \| \| **Occupational classification** \| A&B \| 124 (34.3) \| 126 (34.0) \| 250 (34.2) \| \| C1&C2 \| 120 (33.2) \| 124 (33.4) \| 244 (33.3) \| \| D&E \| 117 (32.4) \| 121 (32.6) \| 238 (32.5) \| \| **Household Income** \| Up to £15,499 per year \| 83 (23.0) \| 96 (25.9) \| 179 (24.5) \| \| £15,500- £24,999 per year \| 83 (23.0) \| 94 (25.3) \| 177 (24.2) \| \| £25,000- £39,999 per year \| 107 (29.6) \| 88 (23.7) \| 195 (26.6) \| \| £40,000 or more per year \| 81 (22.4) \| 84 (22.6) \| 165 (22.5) \| \| Don't know / Prefer not to say \| 7 (1.9) \| 9 (2.4) \| 16 (2.2) \| \| **Education** \| No qualifications, GCSE D-G grades, or Level 1 NVQ \| 69 (19.1) \| 72 (19.4) \| 141 (19.3) \| \| GCSE A*-C grades, or Level 2 NVQ \| 57 (15.8) \| 71 (19.1) \| 128 (17.5) \| \| A/AS level, or Level 3 NVQ \| 62 (17.2) \| 62 (16.7) \| 124 (16.9) \| \| Degree or Professional Diploma \| 152 (42.1) \| 145 (39.1) \| 297 (40.6) \| \| Other / Prefer not to say \| 21 (5.8) \| 21 (5.7) \| 42 (5.7) \| \| **BMI** \| Not Overweight or Obese (Under 25) \| 135 (37.4) \| 165 (44.5) \| 300 (41.0) \| \| Overweight (25- 30) \| 149 (41.3) \| 104 (28.0) \| 253 (34.6) \| \| Obese (30+) \| 77 (21.3) \| 102 (27.5) \| 179 (24.5) \| |
| --- | --- | --- | --- | --- | --- | --- | --- | --- | --- | --- | --- | --- | --- | --- | --- | --- | --- | --- | --- | --- | --- | --- | --- | --- | --- | --- | --- | --- | --- | --- | --- | --- | --- | --- | --- | --- | --- | --- | --- | --- | --- | --- | --- | --- | --- | --- | --- | --- | --- | --- | --- | --- | --- | --- | --- | --- | --- | --- | --- | --- | --- | --- | --- | --- | --- | --- | --- | --- | --- | --- | --- | --- | --- | --- | --- | --- | --- | --- | --- | --- | --- | --- | --- | --- | --- | --- | --- | --- | --- | --- | --- | --- | --- | --- | --- | --- | --- | --- |

### Table S2: Mean score (s.d.) for eating motivations, implicit and explicit liking and perceived attributes by income group and gender

|  | *Males* | | | | *Females* | | | |
| --- | --- | --- | --- | --- | --- | --- | --- | --- |
|  | *Below £15.5K* | *£15.5K- £25K* | *£25K - £39K* | *£40K+* | *Below £15.5K* | *£15.5K- £25K* | *£25K - £39K* | *£40K+* |
| TEMS: Liking | 4.37  (0.73) | 4.65  (0.94) | 4.62  (0.65) | 4.52  (0.68) | 4.64  (0.74) | 4.63  (0.77) | 4.66  (0.71) | 4.69  (0.83) |
| TEMS: Habit | 3.88  (0.82) | 4.02  (1.02) | 3.95  (0.81) | 3.87  (0.92) | 4.02  (0.95) | 4.00  (0.89) | 3.97  (0.82) | 3.94  (0.85) |
| TEMS: Need/hunger | 3.80  (0.70) | 3.85  (0.77) | 3.92  (0.74) | 3.77  (0.86) | 4.01  (0.78) | 4.00  (0.81) | 4.10  (0.76) | 3.93  (0.82) |
| TEMS: Health | 2.94  (1.19) | 3.02  (1.11) | 3.17  (1.19) | 3.33  (1.00) | 3.54  (1.13) | 3.51  (1.12) | 3.66  (1.02) | 3.68  (1.17) |
| TEMS: Convenience | 3.60  (1.01) | 3.66  (0.98) | 3.51  (0.85) | 3.50  (0.89) | 3.86  (0.89) | 3.52  (1.03) | 3.57  (0.79) | 3.63  (0.91) |
| TEMS: Pleasure | 3.34  (0.92) | 3.59  (0.91) | 3.66  (0.88) | 3.53  (0.66) | 3.63  (0.83) | 3.76  (0.67) | 3.66  (0.91) | 3.74  (0.87) |
| TEMS: Price | 3.41  (1.13) | 3.18  (1.16) | 2.96  (1.03) | 2.84  (0.80) | 3.65  (1.13) | 3.36  (1.09) | 3.13  (1.10) | 2.85  (0.88) |
| TEMS: Weight control | 2.27  (1.35) | 2.27  (1.38) | 2.73  (1.36) | 2.78  (1.10) | 3.06  (1.28) | 3.24  (1.26) | 3.38  (0.98) | 3.06  (1.28) |
| Implicit Liking | 0.38  (0.71) | 0.31  (0.57) | 0.41  (0.63) | 0.44  (0.64) | 0.36  (0.69) | 0.55  (0.67) | 0.43  (0.62) | 0.41  (0.70) |
| Explicit Liking | 1.45  (1.02) | 1.50  (0.92) | 1.47  (0.94) | 1.51  (0.92) | 1.70  (0.89) | 1.74  (0.95) | 1.73  (0.87) | 1.48  (1.01) |
| Perceived Healthiness | 2.14  (0.65) | 2.11  (0.66) | 2.13  (0.68) | 2.19  (0.55) | 2.35  (0.65) | 2.42  (0.64) | 2.38  (0.52) | 2.30  (0.58) |
| Perceived Satiety | 0.69  (0.75) | 0.42  (0.80) | 0.63  (0.81) | 0.35  (0.78) | 0.35  (0.85) | 0.37  (1.00) | 0.38  (0.91) | 0.54  (0.93) |
| Perceived Value for Money | 0.84  (1.02) | 0.88  (0.92) | 1.04  (0.81) | 1.04  (0.75) | 1.15  (1.04) | 1.18  (0.92) | 1.14  (0.91) | 0.80  (0.86) |

*TEMS: The Eating Motivations Survey subscales; SC-IAT: Single Category Implicit Association Task scores*

### Table S3: Correlations between implicit and explicit liking and perceived attributes

|  | Implicit Liking | Explicit Liking | Perceived Healthiness | Perceived Satiety | Perceived Value for Money |
| --- | --- | --- | --- | --- | --- |
| Implicit Liking | 1.00 |  |  |  |  |
| Explicit Liking | 0.28 | 1.00 |  |  |  |
| Perceived Healthiness | 0.08 | 0.40 | 1.00 |  |  |
| Perceived Satiety | 0.02 | 0.17 | -0.03 | 1.00 |  |
| Perceived Value for Money | 0.13 | 0.39 | 0.42 | 0.12 | 1.00 |

### Figure S1: Frequency of consumption of (a) cake and (b) cheese by income group

1. Cake
2. Cheese

### Figure S2: Probability (odds ratios) of consuming fruit daily, by (a) education group and gender, and (b) occupational group and gender

1. Education Group and Gender
2. Occupational Group and Gender

### Figure S3: Ratings of: (a) perceived satiety and (b) perceived value for money by education group (adjusted means, with 95% CIs; possible score range -3 to 3)

1. Perceived Satiety
2. Perceived Value for Money
